# *Sciadopitys verticillata* Resin: Volatile Components and Impact on Plant Pathogenic and Foodborne Bacteria

**DOI:** 10.3390/molecules24203767

**Published:** 2019-10-19

**Authors:** David I. Yates, Bonnie H. Ownley, Nicole Labbé, Joseph J. Bozell, William E. Klingeman, Emma K. Batson, Kimberly D. Gwinn

**Affiliations:** 1 Entomology and Plant Pathology, University of Tennessee, Knoxville, TN 210095, USA; yatesd@wcde.org (D.I.Y.); bownley@utk.edu (B.H.O.); emmabat@MIT.EDU (E.K.B.); 2 Center for Renewable Carbon, University of Tennessee, Knoxville, TN 210095, USA; nlabbe@utk.edu (N.L.); jbozell@utk.edu (J.J.B.); 3 Plant Sciences, University of Tennessee, Knoxville, TN 210095, USA; wklingem@utk.edu

**Keywords:** Sciadopitys, resin, *Pseudomonas*, Xanthomonas, Bacillus, *E. coli*, Erwinia, biopesticide, pinene

## Abstract

*Sciadopitys verticillata* (*Sv*) produces a white, sticky, latex-like resin with antimicrobial properties. The aims of this research were to evaluate the effects of this resin (*Sv* resin) on bacterial populations and to determine the impact of its primary volatile components on bioactivity. The impact of sample treatment on chemical composition of *Sv* resin was analyzed using Fourier transform infrared spectroscopy (FTIR) coupled with principal component analysis. The presence and concentration of volatiles in lyophilized resin were determined using gas chromatography/mass spectrometry (GC/MS). Changes in bacterial population counts due to treatment with resin or its primary volatile components were monitored. Autoclaving of the samples did not affect the FTIR spectra of Sv resin; however, lyophilization altered spectra, mainly in the CH and C=O regions. Three primary bioactive compounds that constituted >90% of volatiles (1R-α-pinene, tricyclene, and β-pinene) were identified in *Sv* resin. Autoclaved resin impacted bacterial growth. The resin was stimulatory for some plant and foodborne pathogens (*Pseudomonas fluorescens*, *P. syringae*, and *Xanthomonas perforans*) and antimicrobial for others (*Escherichia coli*, *Bacillus cereus*, *Agrobacterium tumefaciens*, and *Erwinia amylovora*). Treatment with either 1R-α-pinene or β-pinene reduced *B. cereus* population growth less than did autoclaved resin. The complex resin likely contains additional antimicrobial compounds that act synergistically to inhibit bacterial growth.

## 1. Introduction

Control of bacteria that cause plant disease and crop or food spoilage is necessary to ensure a safe, plentiful food supply [1,2,3]. Bioactive natural products from plants are potential sources of new antimicrobials that may also serve as platform compounds for the synthesis of new antibiotics [4] and as biopesticides [5,6]. Dependable, renewable sources are needed to fully develop plant-based products for biopesticide production. Perennial plants can provide substantial amounts of harvestable products each year, thus ensuring a steady supply of biomass for producers, processors, and consumers. Resins from perennial plants have the potential to be renewable sources of antimicrobial agents for use in agriculture and food safety.

Japanese Umbrella Tree, *Sciadopitys verticillata* (Thunb.) Siebold and Zuccarini (Sciadopityaceae), is a needled evergreen that is the sole extant member of the Sciadopityaceae family, a taxonomic group basal to modern conifers [7,8]. Although the native range is limited to small and disjunct populations in Japan, *S. verticillata* is commercially propagated and has been distributed throughout the world [7]. Specimens of the plant are under-utilized in landscapes, in part due to their slow growth and high consequent value. Needles retain their green color year-round, and mature plants retain a pleasing shape with almost no need for maintenance pruning. The species is well adapted to climatic and soil conditions found across the southern Appalachian Mountain region [9]. The tree produces a white, viscous, sticky latex-like resin (Supplementary Figure S1) that seals mechanical wounds and may protect against pathogens and insects [10]. Resin of *S. verticillata* (*Sv* resin) is a complex mixture of solids and liquids that also contains volatile components. *Sv* resin is thought to be the source of Baltic amber [8,11,12]. 

Because it is a renewable resource, *Sv* resin is attractive as a potential source of antimicrobial compounds. When applied directly to a culture of bacteria, drops of unprocessed *Sv* resin have eliminated growth of several bacterial species. Resin collected from plants in summer had strong antibacterial activity against all tested human-associated bacteria except *Escherichia coli*, strong activity against two foodborne pathogens (*Bacillus cereus* and *B. subtilis*), and no activity against plant pathogens (*Agrobacterium tumefaciens*, *Pseudomonas syringae*, *Xanthomonas sp.*, or *Erwinia amylovora*). Subsequently, activity against *E. coli* was noted for resin collected in winter (Yates, personal observation) [9]. This method, while effectively demonstrating the potential of the resin, is insufficient for quantification of activity and for testing of potential antimicrobial components. The work reported here documents differential (antibacterial and stimulatory) effects of *Sv* resin on plant and foodborne pathogenic bacteria and identifies volatiles associated with the resin. 

## 2. Results

### 2.1. Types of Resin

The *Sv* resins used in this study were collected from *S. verticillata* stems or needles (Supplementary Figure S1). If not evaluated immediately, resin samples were stored at −20 °C until use. Resins were classified into three types based on collection date and storage time:

1. Summer: collected in summer and evaluated within 72 h,
2. Summer-Stored: collected in the summer and stored for 6 months before evaluation, and
3. Winter: collected in winter and evaluated within 72 h.

### 2.2. Effects of Treatment on Resin Chemical Fingerprint 

**Figure 1:**
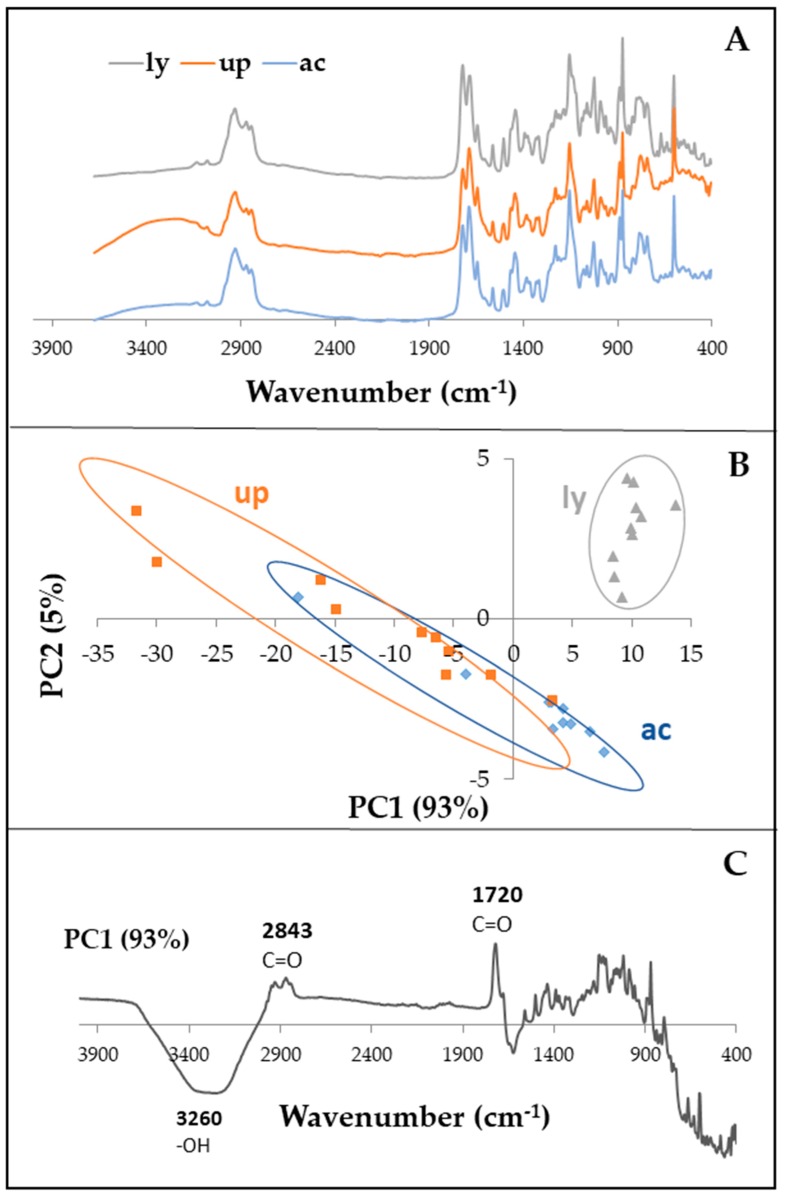
Effects of treatment on resin collected in summer from *Sciadopitys verticillata*: (**A**) Fourier transform infrared (FTIR) spectra of lyophilized (ly; grey), unprocessed (up; orange), and autoclaved (ac; blue) resin. (**B**) Scores plot of the first two principal components obtained by principal component analysis (PCA) of the FTIR spectra of lyophilized (grey triangles), unprocessed (orange squares), autoclaved (blue circles), and *Sciadopitys verticillata (Sv)* resin. (**C**) Loadings plot of PC1.

When centrifuged, freshly extracted *Sv* resin formed a white pellet that was soluble in dimethyl sulfoxide (DMSO), however it was only partially soluble in polar solvents (acetone, ethanol, and methanol). Due to the complexity of the Fourier transform infrared (FTIR) spectra, principal component analysis (PCA), a multivariate method, was used to compare the chemical fingerprints (FTIR) of the resins. In PCA, samples with similar FTIR spectra are plotted close to each other while samples with different chemical compositions are scattered far from each other in the scores plot (Figure 1B). The FTIR spectra of Summer resins collected in the 4000 to 500 cm^−1^ range revealed that there were no significant chemical differences between the untreated (unprocessed) and the autoclaved *Sv* resins (Figure 1). However, a separation between unprocessed/autoclaved and lyophilized resins was observed, demonstrating that lyophilization altered the chemical composition of the resulting resins (Figure 1B). The PC1 loadings plot from the PCA is shown in Figure 1C. Wavenumbers with large loadings, both positive and negative, are those that contribute most to the variation in the scores plot along PC1. The loadings plot identified the wavenumbers that were primary contributors to differences between unprocessed resin and lyophilized resin. Wavenumbers with the greatest loadings were in the -OH, CH, and C=O regions (Figure 1C). In addition, some variability was found within the lyophilized samples, as these samples were scattered along PC1 in the negative quadrant of the scores plot (Figure 1B). Approximately 93% of the variance of the data was explained by the first component (PC1) (Figure 1). Because autoclaving did not alter the FTIR chemical fingerprint (Figure 1B) of the resulting resin when compared to the unprocessed materials, autoclaved resin suspension was used in all antimicrobial tests.

### 2.3. Gas Chromatography/Mass Spectrometry (GC/MS) Analysis of Resin

**Figure 2:**
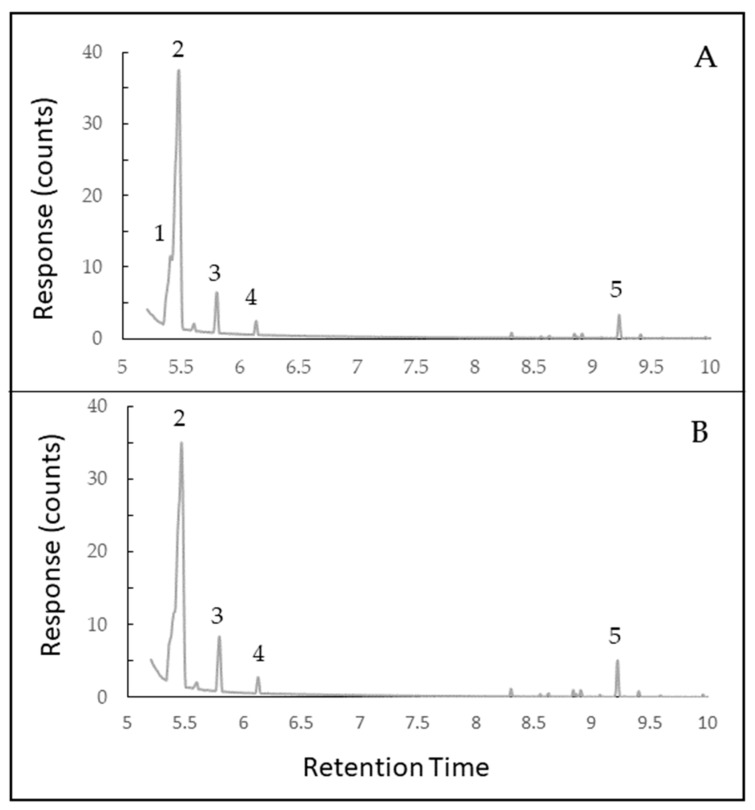
Gas Chromatography (GC) profiles of lyophilized *Sciadopitys verticillata* resin: (**A**) Resin collected in summer and stored at −20 °C for 6 months before evaluation, (**B**) Resin collected in winter and evaluated within 72 h. Compounds with peaks greater than 1% of total peak area: 1 = tricyclene; 2 = 1R-α-Pinene; 3 = β-Pinene; 4 = D-Limonene; and 5 = β-Elemene.

In preliminary tests, bacteria did not grow on a nutrient agar overlay placed over wells in nutrient agar that were filled halfway with autoclaved resin, suggesting that some of the active component(s) were volatile [13]. Eighteen volatilized compounds were identified by gas chromatography/mass spectrometry (GC/MS) of lyophilized resins collected in Summer-Stored and Winter resins, with few seasonal differences among volatile compounds (Table 1; Figure 2). The primary compound in both lyophilized resins (>73% total volatiles) was 1R-α-pinene. Concentration of 1R-α-pinene was calculated to be 0.27 mg/g in Summer-Stored resin and 0.34 mg/g in Winter resin, based on a calibration curve (Supplementary Figure S1). Both Summer-Stored and Winter resins contained β-pinene (6% to 8% of volatiles). In Summer-Stored resin, the second most abundant compound (17% of total peak area) was tricyclene (Table 1). Tricyclene was below the level of detection in Winter resin and was not found in *Sv* resin collected in winter from another tree [13].

### 2.4. Impact of Sciadopitys verticillata Resin on Bacterial Population Counts

Summer and Winter resins were evaluated for impact on bacterial populations for all seven bacteria listed in Table 2. Activity of resins was classified based on antimicrobial effectiveness tests [14]. Activity was categorized into three levels based on the degree of difference (x) between the response of the no-resin control and that of the 50 μL dose of resin suspension:

1. Strong antimicrobial/stimulation response: x > 1 log_10_,
2. Mild antimicrobial/stimulation response: x < 0.5 log_10_,
3. Moderate antimicrobial/stimulation response: 0.5 log_10_ < x < 1 log_10_.

**Figure 3:**
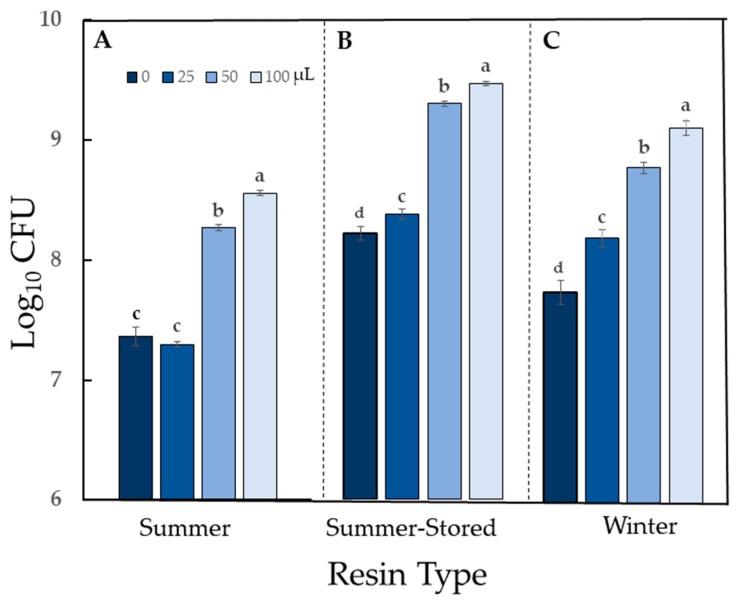
Growth stimulation of *Pseudomonas fluorescens* with autoclaved *Sv* resin: (**A**) Resin collected in summer and evaluated within 72 h. (**B**) Resin collected in summer and stored at –20 °C for 6 months before evaluation. (**C**) Resin collected in winter and evaluated within 72 h. Treatment doses (0, 25, 50, and 100) are volumes of suspended resin (μL) in a total volume of 300 μL. For each resin type, bars with the same letter are not different according to Tukey’s test at *P* < 0.0001. Data shown are arithmetic means + standard errors.

Activity of autoclaved *Sv* resin was bacterial species-dependent and ranged from strong stimulation to strong antimicrobial activity (Table 2). The impact on growth of all bacteria was significant (*P* < 0.05). Treatment of *Pseudomonas fluorescens* with resin yielded strong population growth stimulation, in a dose-dependent manner for Summer resin and a moderate population growth stimulation for Winter resin (*P* < 0.0001 for both resins) (Figure 3). 

**Figure 4:**
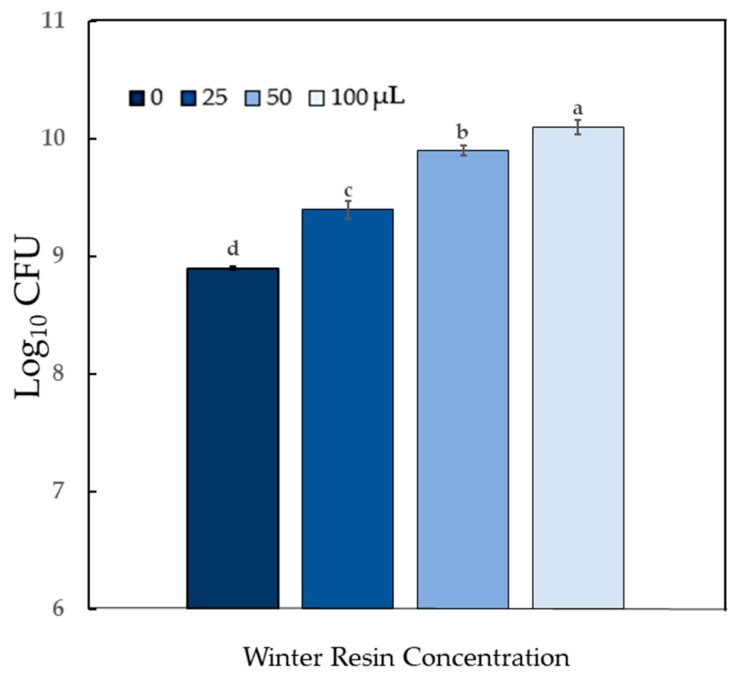
Growth stimulation of *Pseudomonas syringae* pv. *tomato* with autoclaved *Sv* resin. Resin was collected in winter and evaluated within 72 h. Treatment doses (0, 25, 50, and 100) are volumes of suspended resin (μL) in a total volume of 300 μL. Bars with the same letter are not different according to Tukey’s test at *P* < 0.0001. Data shown are arithmetic means + standard errors.

Because *P. fluorescens* was able to use the resin as a food source, it was necessary to evaluate a closely related phyllosphere phytopathogen to determine if it could also utilize the resin. Populations of *Pseudomonas syringae* pv *tomato* (*Pst*), the causal agent of bacterial speck of tomato, increased in a dose-dependent manner similar to *P. fluorescens*, thus validating the stimulatory effect on *P. fluorescens* growth (*P* < 0.0001) (Figure 4). 

**Figure 5:**
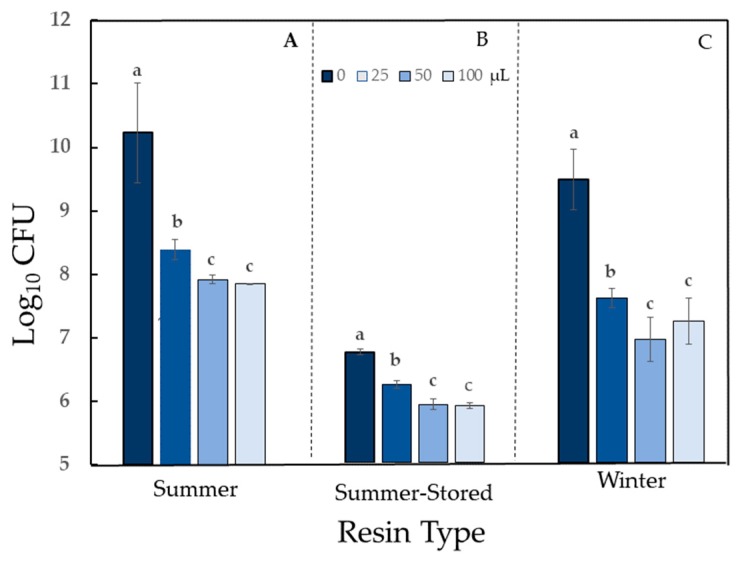
Antimicrobial activity of autoclaved *Sv* resin against *Erwinia amylovora*. (**A**) Resin collected in summer and evaluated within 72 h. (**B**) Resin collected in summer and stored at –20 °C for 6 months before evaluation. (**C**) Resin collected in winter and evaluated within 72 h. Treatment doses (0, 25, 50, and 100) are volumes of suspended resin (μL) in 300 μL. For each resin type, bars appearing with the same letter are not different according to Tukey’s test (*P* < 0.05). Data shown are arithmetic means + standard errors.

The antimicrobial response of *Erwinia amylovora* (the bacterial phytopathogen that causes fire blight of apples and other plants in the family Rosaceae) was strong at low doses for both Summer and Winter resins (significant difference among treatments for both resins at *P* < 0.0001) (Figure 5). All tested resins had a mild impact on two additional phytopathogens: *Xanthomonas perforans*, causal agent of diseases on many solanaceous crops (mild growth stimulation), and *Agrobacterium tumefaciens*, the bacterial phytopathogen that causes crown gall (mild inhibition) (Table 2; Supplementary Figure S3). 

**Figure 6:**
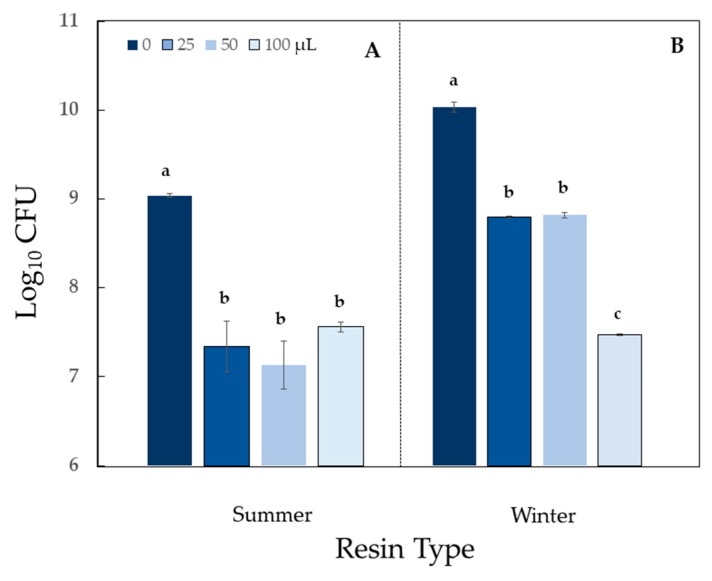
Antimicrobial activity of autoclaved *Sv* resin against *Bacillus cereus*. Resins collected in (**A**) summer or (**B**) in winter were tested within 72 h. Treatment doses (0, 25, 50, and 100) are volumes of suspended resin (μL) in 300 μL. For each resin type, bars appearing with the same letter are not different according to Tukey’s test (*P* < 0.05). Data shown are arithmetic means + standard errors.

*Bacillus cereus*, a common cause of food poisoning in humans, was sensitive to Summer resin (exhibited strong antibiosis) (*P* < 0.0001), whereas, the response to Winter resin was classified as moderate (*P* < 0.0001) (Table 2; Figure 6). One additional common food poisoning agent, *Escherichia coli, Sv* resin had only a mild inhibitory effect (Table 2; Supplementary Figure S4). 

Storage at −20 °C for six months had no effect on the activity category of resin against *E. coli* or *P. fluorescens* (Table 1; Figure 3B; Supplementary Figure S4). Activity against *Erwinia* was reduced from high (Summer) to moderate (Summer-Stored) antibiosis (Table 1; Figure 5B). 

### 2.5. Antibacterial Activity of α- and β-pinene

Based on GC/MS analysis, concentration of α-pinene, the predominant volatile in *Sv* resin, was estimated to be 2.0 mM in the 100 µL dose of resin suspension, and this concentration was used as a basis for assigning treatments in antibacterial assays. Because no zones of inhibition were observed in disc diffusion tests even at concentrations of α-pinene that were 3 × 10^3^ times greater than estimated for lyophilized resin, assays were performed to monitor growth by quantifying increases in sample turbidity by spectral absorbance.

**Figure 7:**
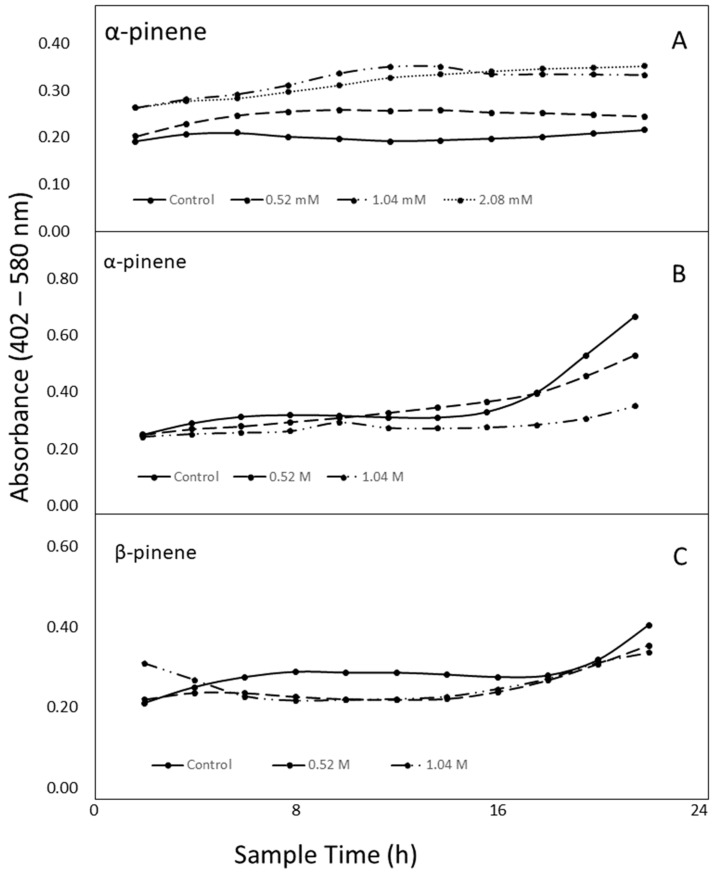
Changes in absorbance of *Bacillus cereus* cultures exposed to pinenes. (**A**) Concentrations of α-pinene lower than or equal to estimates of α-pinene concentration in *Sv* resin. (**B**) Concentrations of α-pinene of up to 1000 times greater than estimates of α-pinene concentration in *Sv* resin. (**C**) Concentrations of β-pinene up to 1000 times greater than estimates of α-pinene concentration in *Sv* resin. Wide-band filter (402–580) was used to measure turbidity increase associated with growth of bacterial cultures.

In experiments where the impact of pinene (<2.0 mM) on bacterial population growth was monitored across time by absorbance, there was a significant treatment × time interaction (*P* = 0.0001). Populations of *B. cereus* increased after 8 h in all α-pinene treatments that corresponded to low, moderate, or high resin doses used to evaluate activity (see previous section). Maximum growth was observed at 12 h (4- to 8-fold increase) (Figure 7A). The effect of α-pinene concentrations on bacteria as measured by colony forming units (CFU) was also tested at mM concentrations used in the growth curve studies, and population counts were 50% greater than control after a 24 h exposure to low concentration of α-pinene. Growth was increased by approximately two-fold in moderate and high α-pinene treatments.

Because lyophilization likely reduced the most volatile constituents in *Sv* resins, concentration of α-pinene in the resin may have been considerably higher than the estimated 2.0 mM. Thus, we tested the compound at molar concentrations. In this experiment, there was also a significant treatment × time interaction (*P* < 0.0001). In the high concentration (2.08 M, approximately 100-fold greater than estimated), α-pinene reacted with the plastic of the plates used in the experiment and altered transparency to the extent that spectroscopic measurements were rendered inaccurate. The other tested molar concentrations of α-pinene were approximately 250 times (0.52 M, low) and 500 times (1.04 M, moderate) greater than estimates for the lyophilized resin (Figure 7B). Growth of *B. cereus* (as measured by absorbance) was inhibited by both doses for 16 h (*P* < 0.0001), after which, the lower dose was not significantly different from the control (Figure 7B). β-pinene did not react with the plastic at 2.08 M so activity of β-pinene against *B. cereus* was tested at four molar concentrations. There was also a significant treatment × time interaction (*P* < 0.0001) in treatments with β-pinene. Changes in populations of *B. cereus* in response to treatment with β-pinene were only different in wells sampled at 8 and 12 h (Figure 7C). 

The effect of α-pinene concentrations on bacteria as measured by colony forming units (CFU) was tested at molar concentrations used in the growth curve studies. There was a reduction in *B. cereus* counts at all concentrations as compared to control. CFUs were reduced by 21% in the lowest molar concentration (0.5 M) and 53% in the highest molar concentrations of α-pinene. The moderate concentration treatment reduced populations significantly more than the low concentration (*P* < 0.0094).

## 3. Discussion

Bioactivity of *Sv* resin is most likely explained by activities conveyed by a complex mixture of volatile and nonvolatile compounds. Volatiles were thought to contribute significantly to resin bioactivity because in our preliminary tests, bacteria did not grow on a medium overlaid directly above wells containing autoclaved resin [13]. Because removal of water was necessary for analysis of resin using GC/MS, lyophilized resin was used to identify volatile components even though lyophilization did impact composition. Lyophilized leaves of stevia (*Stevia rebaudiana*) had been reported to contain 74% less α-pinene and 40% less limonene than fresh samples [15]. The majority of the eighteen identified volatile compounds in our resin were monoterpenes (92–95%) and sesquiterpenes (3–5%). Vapor pressures of known compounds that constituted more than 1% of the total volatiles were greater than those that were present in lower amounts. High concentrations of terpenes in *Sv* resin were not surprising because terpenes are typically abundant in conifer resins. Resins also contain mixtures of phenolics, alkaloids, minerals, and carbohydrates [16]. 

Approximately 95% of total *Sv* resin volatiles was due to three compounds. The principal constituent of both *Sv* Summer-Stored and Winter resins was the terpene, 1R-α-pinene. Both resins also contained β-pinene. Although tricyclene was present in Summer-Stored resin, no tricyclene was detected in resins collected in the Winter. In general, there were few differences between Summer and Winter resins. Direct evidence that tricyclene is inhibitory to *B. cereus* is lacking due to the fact that this compound is not commercially available. We are unaware of any report on the antimicrobial activity of the pure compound. There is indirect evidence that tricyclene may play a role in seasonal differences in activity of the resin against *B. cereus*, the only Gram-positive (G+) species tested in this study. The essential oil of *Cordia verbenacea*, in which tricyclene was the predominant volatile, was active against G+, but not Gram-negative (G-) bacteria [17]. 

Two species in the genus *Pseudomonas* had similar reactions to *Sv* resin. Stimulation of *P. fluorescens* by the *Sv* resin called into question the validity of using the resin as a biopesticide; therefore, we also tested *P. syringae* pv. *tomato*, a plant pathogen that causes reduced fruit quality and post-harvest storage and for which control by terpenes has been evaluated [18]. Treatment of *Pst* with Winter resin resulted in a dose-dependent response similar to that of *P. fluorescens*. Species of *Pseudomonas* were the most prevalent resin-tolerant bacteria isolated from the resin of Scotch pine (*Pinus sylvestris*) and from galls induced on Scotch pine by larvae of *Retinia resinella* moth, therefore the stimulatory effect of *Sv* resin on species of *Pseudomonas* is not unexpected [19]. Although pinenes are antimicrobial compounds that are prone to skeletal rearrangements and interfere with the form and/or function of cell membranes [20,21], some bacteria, including strains of *P. fluorescens*, biodegrade α-pinene and use it as a carbon source [22]. Thus, metabolism of pinene may have contributed to the increased population counts of *P. fluorescens* and *Pst* in resin treatments. Treatment with α-pinene has been reported to influence symbiotic bacteria community diversity of pinewood nematode (*Bursaphelenchus xylophilus*). Ratios of symbiotic *Pseudomonas* spp. increased from 16% to 40% when the nematode was treated with α-pinene [23]. 

Although both members of the Enterobacteriaceae were inhibited, *Sv* resin was more active against *Erwinia amylovora* (strong antibiosis) than *E. coli* (mild antibiosis). Growth of both bacteria has been reported to be was reduced by pinenes, but they differ in sensitivity. Concentrations of α-pinene and β-pinene of at least 1500 mg/L (11 mM) were required to inhibit growth of *E. amylovora* [24] and the minimum inhibitory concentration against *E. coli* O157:H7 was 2% (147 mM) [20]. However, the enantiomeric form was not identified in either study and these are known to influence antimicrobial activity. For example, (+)-α-Pinene had moderate activity against *E. coli* (G-) whereas (−)-α-pinene had no activity [25]. In our study, all three resin types were moderately active against *E. coli*. The antimicrobial response was mild when cultures were treated with Winter resin even though Winter resin had slightly greater concentrations of both α-pinene and β-pinene than Summer-Stored resin (moderate activity). In a previous study, pelleted resin collected in the summer and applied directly to bacterial cultures did not inhibit the growth of *E. coli* [9]; thus, it is likely that the direct application method used in that study can only be used to identify strong antibiosis. The antibiotic effect against *E. amylovora* was strong at all doses of both Summer and Winter resins. Some strains of *P. fluorescens* have been used as biological control agents for disease control of fire blight of apples and pears caused by *E. amylovora* [26]. *Sv* resin or its components have the potential to be used in control of fire blight disease in combination with *P. fluorescens* as a biocontrol organism; however, careful testing should be performed to evaluate the impact of the resin on diseases of apples caused by pathovars of *P. syringae*. 

Members of the genus, *Bacillus*, are characterized by aerobic or facultatively aerobic growth and formation of dormant endospores [27]. The *B. cereus* group includes *B. cereus* and ten additional closely related species from diverse natural environments, including *B. anthracis* (causal agent of anthrax in humans and animals) and *B. thuringiensis* (an entomopathogen) [28]. Control of the members of the *B. cereus* group that cause food spoilage is difficult, they can enter food production and processing operations at various points [28]. In our study, *B. cereus* was chosen for evaluation of antibacterial compounds because it has emerged as a contaminant in food products, many antibiotics are not useful for treatment, and natural controls are sought in the food industry. All strains isolated from Tunisian food projects harbored one or more non-hemolytic enterotoxin (*nhe*) genes, and approximately 60% had hemolytic enterotoxin (*hbl*) genes. Strains were resistant to some antibiotics: ampicillin (90.8%), novobiocin (88%), tetracycline (5.7%), vancomycin (4%), erythromycin (1.2%), and streptomycin (1.1%). All strains were sensitive to rifampicin and ciprofloxacin [29]. In Canada, *B. cereus* and its enterotoxins were found in pasteurized milk tested at its best-before date. In products stored at 7 °C, 5.5% of tested products had *B. cereus* at 10^5^ CFU/mL, and about 4% contained enterotoxins at a level that may result in foodborne illness. At 10 °C, these numbers increased to 31% with 10^5^ CFU/mL, and all positive samples contained enterotoxins at harmful levels. For products stored at 4 °C, counts of 10^5^ were found in less than 1% of the samples [30]. In our study, treatment with α-pinene increased the growth of *B. cereus* at concentrations estimated to be in the 100 µL dose of *Sv* resin (2.0 mM), but both α- and β-pinene were antimicrobial at concentrations greater than 0.5 M. 

Many essential oils that contain pinene have activity against bacteria in the *B. cereus* group, but to our knowledge, this is the first report in which low concentrations of the compounds stimulate bacterial growth, a potential problem if dilute solutions are used to clean food preparation surfaces. Other species of *Bacillus* or closely related genera can utilize pinene; however, terpene is inhibitory to others. At concentrations of up to 15 mM, α-pinene, β-pinene, or limonene were degraded by *B. pallidus* [31], currently classified as *Aeribacillus pallidus* [32], but a strain of *B. simplex* (distantly related to *B. cereus* [27]), that was isolated from a mountain pine beetle, *Dendroctonus ponderosae*, was inhibited by 1% α-pinene [33]. 

## 4. Materials and Methods 

Due to the high value of individual trees, collections for antimicrobial testing were limited to *Sv* resin from a single tree (Laurels Nursery, Elizabethton, TN, USA). The source tree was propagated by a stem cutting from a tree purchased in Canby, OR in 1990 and was extensively pruned across a period of at least 10 years. The tree was grown in full sun. Granular fertilizer (10N-10P-10K) was applied twice a year to the soil surface at the dripline. The tree was not irrigated. Pesticides were not applied during the study period or six years prior. Resins were collected both in summer (June/July) and in winter (February/March), then stored at −20 °C.

### 4.1. Effects of Treatment on Resin Chemical Fingerprint 

A combination of FTIR and PCA provided a fast and reliable method for determining effects of experimental treatments on *Sv* resin chemical fingerprint. The *Sv* resin was collected from freshly cut ends of stems or from bundles of needles (8–12/bundle) placed in sterile deionized water (approximately 0.5 mL) for 1 h (Supplementary Figure S1). Suspensions were centrifuged (7800× *g*) for 5 min and supernatant removed. Pellets of *Sv* resin were either frozen (−20 °C; 48 h) and then lyophilized (−40 °C; <0.133 mBars; 72 h) (FreeZone, Labconco, Kansas City, MO, USA) for 72 h or were suspended in distilled water for a final concentration of 33% resin/resin suspension (*v/v*) and autoclaved. All samples were scanned using a Thermo Nicolet Nexus Model 670 FTIR spectrometer equipped with a Golden Gate MKII Single Reflection Attenuated Total Reflectance (ATR) accessory (ThermoFisher, Norcross GA, USA). Scans were collected in the 4000–500 cm^−1^ spectral range with 8 cm^−1^ spectral resolution, and 32 scans were averaged for each spectrum. Spectra used for PCA included five to ten independently expressed and scanned subsamples. Multivariate analyses of the resin FTIR spectra were performed using Camo Unscrambler (version 8.0) (Camo Analytics, Magnolia, TX, USA) to observe differences and groupings between the sample sets (Scores plot) after mean normalization and multiplicative scatter correction of the data. Major wavenumbers responsible for the grouping were identified from the loadings plots (Figure 1) when a PCA scores plot indicated a difference between samples sets. 

### 4.2. GC/MS Analysis of Resin

The GC/MS data were collected on Winter resins stored for 2–3 days at −20 °C and Summer-stored resins (stored for the 6-month period between collections). This allowed both samples to be processed and analyzed concurrently. Resin pellets, lyophilized as described above, were dissolved in DMSO (100,000 ppm). Once dissolved, samples were diluted to a concentration of 200 ppm with optima grade ethyl acetate. Diluted samples were analyzed using an Agilent Technologies 7890B Gas Chromatograph (Santa Clara, CA, USA) coupled to a 5977 Agilent Mass Selective Detector fitted with a 250 °C splitless inlet by autosampler. A mobile phase of ultra-high purity helium gas was used to carry samples (1 µL) along the 30 m × 0.25 mm (250 micron) column. The ramp was first held at 50 °C for 0.5 min before increasing to 300 °C at a rate of 20 °C/min with a 2-min bake-out at 325 °C. Peaks were identified using MassHunter software equipped with the NIST02 Library. Standard curves were generated for α-pinene and β-pinene. 

Peak area percentages were calculated, and data were analyzed with a Wilcoxon Signed rank test at *P* = 0.05. Data were not normally distributed; therefore, this test was used for the non-parametric format of paired *t*-tests. All data were analyzed for significance with SAS 9.4 TS1M3 for Windows (SAS Institute Inc. Cary, NC, USA). A linear equation based on a standard curve (Supplementary Figure S1) was used to calculate the concentration of α-pinene in Winter and Summer-Stored resins. 

### 4.3. Impact of Sciadopitys verticillata Resin on Bacterial Population Counts

Activity of *Sv* resin against six G- species of bacteria and one G+ bacterial species was tested in this study (Table 1). Plant pathogenic bacteria were obtained from B. H. Ownley. Additional bacteria were purchased from Carolina Biological Supply (Burlington, NC, USA). All bacteria, except *Pst*, were screened to assess impact of both Summer and Winter resins on microbial activity. Resin suspensions were prepared as described above for autoclaved treatments. For most experiments, resin was tested for antibacterial activity within 72 h of collection. Activity of Summer-Stored resin was tested for three bacteria (*E. amylovora, E. coli,* and *P. fluorescens*) (Table 1).

Bacterial cultures were prepared in Difco™ Nutrient Broth (NB) (Becton, Dickinson, Franklin Lakes, NJ, USA) and incubated at 30 °C. After 24 h, cultures were centrifuged for 5 min at 10,000 rpm. Supernatant was removed and the pellet was re-suspended in liquid NB. The suspension was then diluted to a standardized transmittance (75% ± 2.5%) with a Turbidimeter™ (Biolog Inc., Hayward, CA, USA). All treatments were incubated in honeycomb microplates (Growth Curves USA, Piscataway, NJ, USA). NB medium (100 μL) and bacterial suspension (100 µL) were added to each test well. Four resin suspension doses were tested: 0 (control); 25 µL; 50 µL; and 100 µL. Sufficient deionized water was added to each well to bring the final volume to 300 µL. The final resin concentration was 0 to 11% (*v/v*) *Sv* resin. Microplates were incubated in a Labsystems Bioscreen C (Growth Curves USA, Piscataway, NJ, USA) using constant shaking at 30 °C. Photometric monitoring of bacterial population growth was not possible due to the uneven suspension of the resin in an aqueous environment; therefore, microdilution plating [34] was used to determine numbers of colony forming units (CFU) at 24 h. Well contents were serially diluted in sterile water, and five 10 µL aliquots of each dilution (10^−3^ to 10^−8^) were spotted onto Nutrient Agar (Oxoid, Basingstoke, Hampshire, England). Cultures were incubated either at room temperature or 30 °C (*E. coli* only). Colonies were counted after 1 to 2 days. Each microplate well served as a replicate, and each treatment was replicated. Three dilution series were made from each well. Bacteria for which there were mild or no differences with *Sv* treatment in the first trial were not retested. For others, the experiments were repeated twice (n = 3). 

Data were analyzed using SAS (Version 9.4 TS1M3) for Windows (SAS Institute Inc., Cary, NC, USA). Population data were analyzed with mixed model ANOVA. Experiments were arranged in a randomized complete block design (winter data) or completely randomized design (summer data). Data were rank transformed to meet the assumptions of normality and equal variance in ANOVA. Data shown are arithmetic means + standard error. Post hoc multiple comparisons among treatments were conducted with Tukey’s adjustment at *P* = 0.05. 

### 4.4. Antibacterial Activity of α- and β-pinene

Based on GC/MS data and standard curves, concentrations of α-pinene in lyophilized *Sv* resin were determined, and activity of α-pinene against *B. cereus* was tested at or above calculated concentrations. Filter-sterilized (45 µm filter) suspensions of α-pinene (Aldrich Chemical Inc., Milwaukee, WI, USA) were used to saturate autoclaved diffusion discs. Because disc diffusion assays rely on the ability of the compound to move from the disc into the agar and α-pinene has a low solubility in water, concentrations up to 3600 times greater than estimated for the resin (0 (control), 0.5 M, 1.0 M, 2.0 M, and 6.3 M (no dilution)) were tested. Bacterial suspension (1 mL) prepared as described above was sprayed onto Nutrient Agar. After 10 min, diffusion discs were placed onto medium. Cultures were incubated for 48 h, and inhibition zones around discs were then measured. 

Effects of α-pinene on bacterial population counts were determined as described for resin assays, except that α-pinene was substituted for resin and population growth was monitored by absorbance in the wide band filter used to estimate turbidity (420–580 nm). In the first set of experiments, four concentrations, at or below levels estimated in resin, were tested (0 (control), 0.52 mM, 1.04 mM, and 2.08 mM). Experiments were repeated twice.

In the second set of experiments, concentrations of α-pinene and β-pinene more consistent with those used in the disc diffusion assay (up to 10^3^-fold > concentration in lyophilized *Sv* resin) were tested. Final concentrations of the compounds tested in the microplate wells were 0.52 M, 1.04 M, or 2.08 M (α-pinene) and 0.58 M, 1.18 M, or 2.36 M (β-pinene). Assays were performed as described at the lower doses. In duplicate experiments, effects of the molar concentrations of α-pinene on bacterial population counts were determined as described above.

## 5. Conclusions

Control of bacteria that cause food borne illness in humans and plant disease is essential for global food security. Our findings that activity of *Sv* resin is species-dependent and can be either stimulatory or antimicrobial are initial steps in the evaluation of *Sv* resin as a source of natural and sustainable biopesticides. The resin is a complex chemical matrix, and this study documents seasonal differences in resin antimicrobial activities and chemical composition. This is the first report of volatiles from *Sv* resin. Monoterpenes represented greater than 90% of the volatiles collected from *Sv* resin. The primary volatile was α-pinene, tricyclene was the second most abundant volatile from resin collected in Summer but was not found in resin collected in Winter. Population counts of several plant pathogens and food spoilage bacteria were reduced in the presence of the resin. This study is the first documentation of growth stimulation by *Sv* resin. The resin can stimulate the growth of two species of plant pathogens (*Pst* and *X. perforans*) so it can only be used in select cropping systems. For example, this knowledge may be used to develop new biopesticide combinations for control of fire blight caused by *E*. *amylovora*. The resin has strong inhibition of the pathogen and strong stimulation of a biocontrol agent used to control the disease (*P*. *fluorescens*). This study also provides the first evidence that growth of *B. cereus* is stimulated at low concentrations of α-pinene but inhibited at higher concentrations. This study provides new insight into the nature of the stimulatory/antibacterial activity of *Sv* resin, but before this resource can be fully utilized, additional studies are needed. It is unlikely that all antibacterial compounds in the resin are volatile and identified by the GC/MS procedures used in this study. Future research will focus on additional characterization of the resin by techniques such as nuclear magnetic resonance and pyrolysis CGC/MS and on additional uses for the resin. 

## Figures and Tables

**Table 1:** Volatile components of *Sciadopitys verticillata* resins analyzed by gas chromatography/mass spectrometry (GC/MS).

			SUMMER-STORED		WINTER	
Compound ^a^	Retention Time (min)	Vapor Pressure ^b^	Score	% Peak Area	Score	% Peak Area
***Monoterpenes***				94.738		92.232
1R-*α*-Pinene ^c^	5.474	4.75	93	70.677	92	82.003
Tricyclene	5.399	4.45	88	16.313	NP	NP
*β*-Pinene ^c^	5.794	2.93	81	5.394	83	7.656
D-Limonene	6.132	1.98	88	1.570	88	1.784
Camphene	5.600	2.5	81	0.784	80	0.789
***Methane monoterpenes***				*0.855*		*0.703*
3 7 *α*-Terpinyl propionate	8.306	0.013	78	0.415	80	0.579
7 α-Terpinyl propionate	9.588	0.013	63	0.040	69	0.124
***Sesquiterpenes***				*3.193*		*4.925*
*β*-Elemene	9.222	0.0276	94	2.441	94	3.635
*β*-Cubebene	8.907	0.014	82	0.373	85	0.541
*γ*-Cadinene	8.627	0.003	85	0.164	88	0.267
Caryophyllene	8.867	0.300	87	0.109	88	0.195
Copaene	8.558	0.038	82	0.106	79	0.159
*γ*-Cadinene	9.067	0.003	NP	0.000	93.	0.128
***Other***				*1.248*		*2.139*
Contaminant (Siloxane)	10.189	NA	NA	0.796	NA	0.900
Unknown ^d^	8.844	NA	85	0.369	87	0.586
1-Naphthalenol	9.405	NA	77	0.304	78	0.472
*β*-Ionone	9.954	0.054	72	0.104	74	0.181
1,5,9,9-Tetramethyl-1,4,7-cycloundecatriene	9.073	NA	70	0.043	NP	0.000
11-Acetoxy tetracyclododecane	9.845	0.135	61	0.032	NP	0.000

^a^ Resin compounds identified using the MassHunter software to search the National Institute of Standards and Technology library (NIST02) of mass spectra and listed by percent of total peak areas (largest to smallest). Not Present (NP) indicates that the compound was not identified in the resin sample. ^b^ Values are in mm Hg at 25 °C. Data collected from PubChem, ChemSpider, or Good Scents databases (NA = not available). ^c^ Identified using co-injection with standard compounds. ^d^ Unknown compound was identified as β-carene by the software, but the vapor pressure of the compound is inconsistent with the retention time.

**Table 2:** Effect of *Sciadopitys verticillata* resin on populations of tested bacteria.

Family	Pathogen	Economic Importance	Reaction to Resin ^2^		
(Gram stain)	Strain ^1^		Summer	Winter	Summer-Stored
Bacilliaceae (+)	*Bacillus cereus* CB# 154869	foodborne illness	---	--	n.d.
Enterobacteriaceae (-)	*Erwinia amylovora* UTBO# E9	plant pathogen	---	---	--
	*Escherichia coli* CB# 155068	foodborne illness; biotechnology	-	-	-
Pseudomonadaceae (-)	*Pseudomonas fluorescens* CB# 155255	food contaminant; bio-manufacturing; biological control	+++	++	+++
	*Pseudomonas syringae* pv. *tomato* UTBO# 268	plant pathogen	n.d.	+++	n.d.
Rhizobiaceae (-)	*Agrobacterium tumefaciens* UTBO# C58	plant pathogen	-	-	n.d.
Xanthomonadaceae (-)	*Xanthomonas perforans* UTBO# SB1	plant pathogen; bio-manufacturing	+	+	n.d.

^1^ CB strains were purchased from Carolina Biological Supply (Burlington, NC); UTBO = strains from the collection of Dr. Bonnie H. Ownley (University of Tennessee, Knoxville, TN); ^2^ Reaction to resin summarized as the difference in mean population counts (log_10_) between the no-resin control and the moderate dose of *Sciadopitys verticillata* resin suspension (50 µL): (**+**, < 0.5 log increase (mild); **++**, 0.5 log < increase < 1.0 log (moderate); **+++**, > 1.0 log increase (strong); **-**, < 0.5 log decrease (mild); **--**,0.5 log < decrease < 1.0 log (moderate); ---, > 1.0 log decrease (strong); n.d., not determined).

## Supplementary Materials

The following are available online, Figure S1: *Sciadopitys verticillata* resin, Figure S2: Standard curve used to calculate concentration of 1R-α-pinene in resin, Figure S3: Impact of *Sv* resin on growth of phytopathogenic bacteria, *Agrobacterium tumefaciens* and *Xanthomonas perforans*, Figure S4: Antimicrobial activity of autoclaved *Sv* resin against *Escherichia coli*.
