# Supplementary material for: Sciadopitys verticillata Resin: Volatile Components and Impact on Plant Pathogenic and Foodborne Bacteria
Source: Molecules. 2019 Oct 19;24(20):3767. doi: 10.3390/molecules24203767 (PMC6833092; doi:10.3390/molecules24203767)
Supplement: Supplementary file 1 [file molecules-24-03767-s001.zip › molecules-613178-supple/supplementary.docx]

**
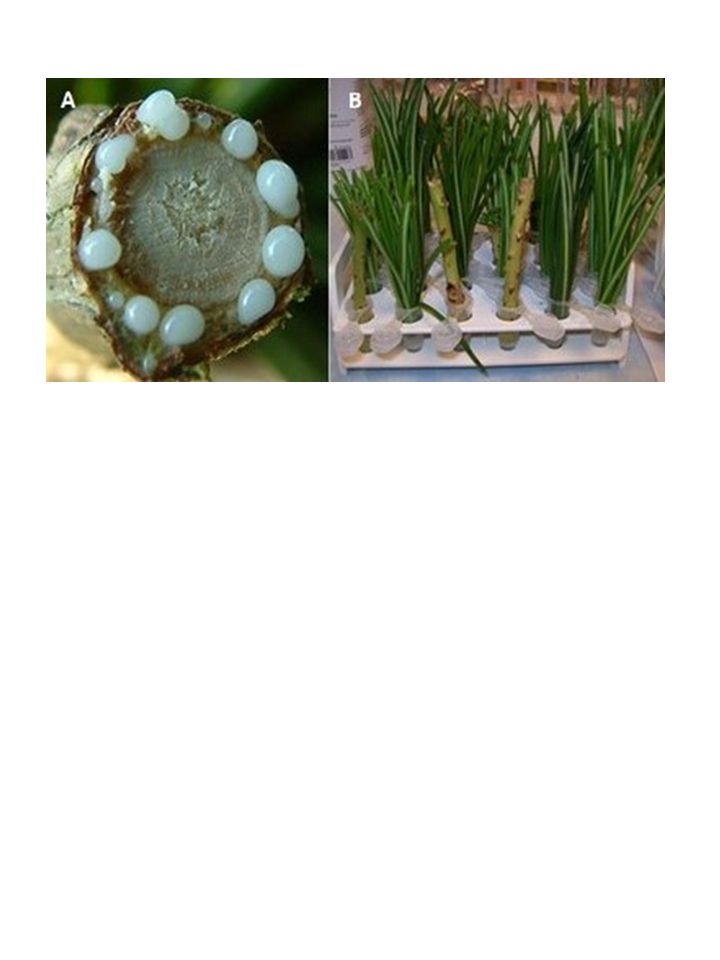
Figure S1.** *Sciadopitys verticillata* resin. (**A**) Resin exuding from cut stem. (**B**) Method used to collect resin from needles.


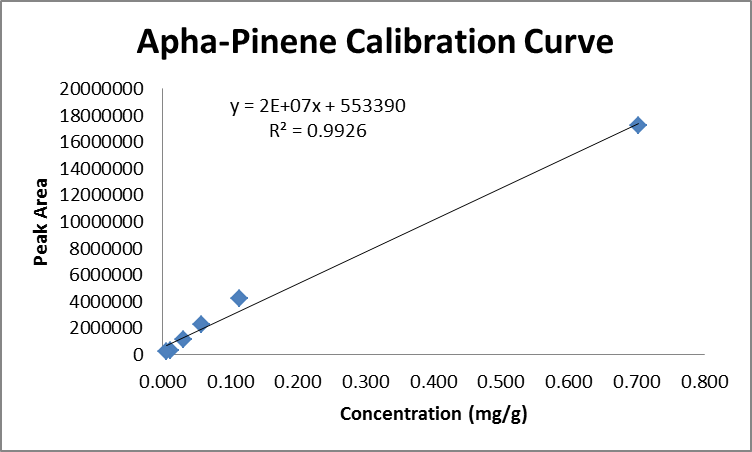
.

**Figure S2.** Calibration curve used to calculate the concentration of 1R-α-Pinene.


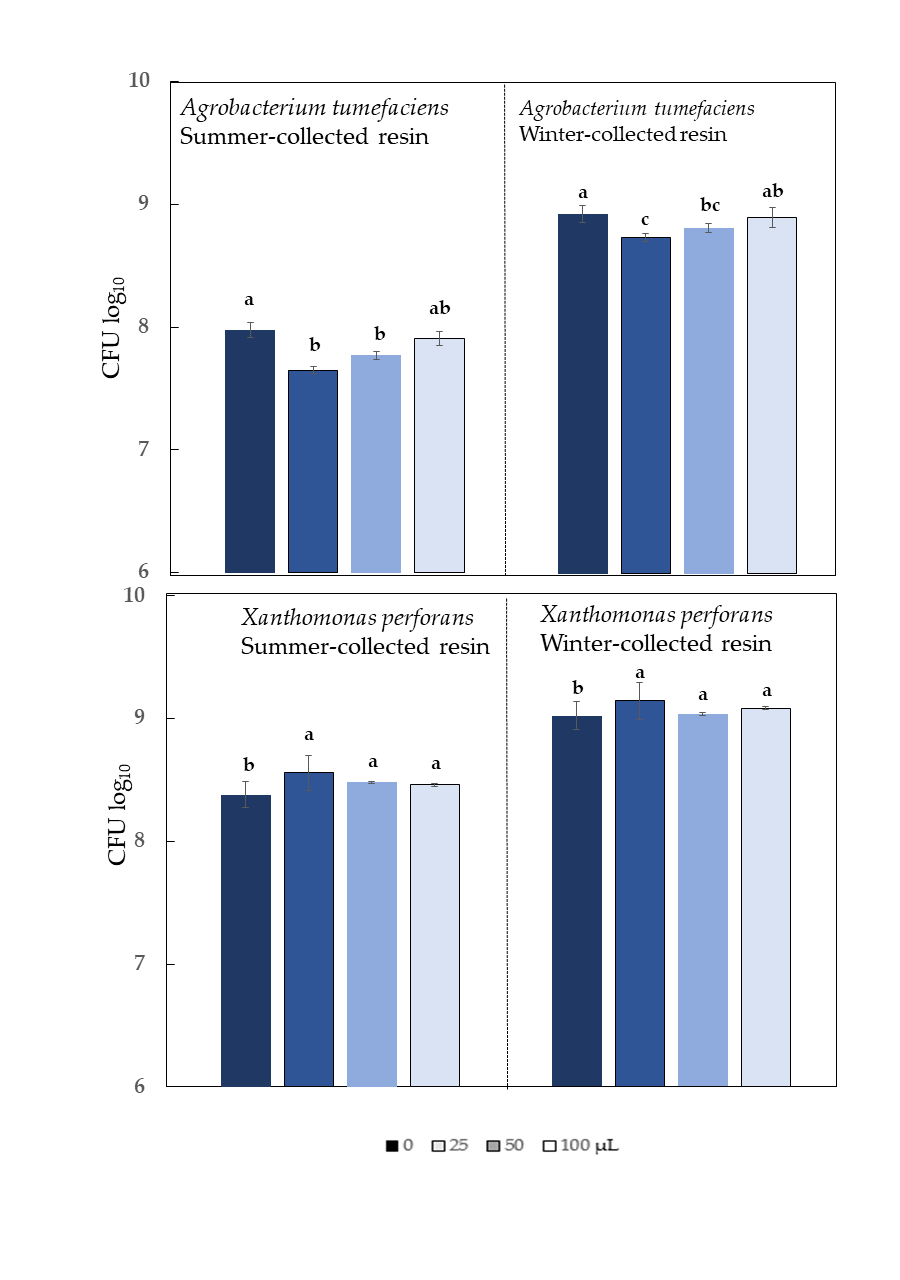
.

**Figure S3.** Impact of *Sv* resin on growth of phytopathogenic bacteria, *Agrobacterium tumefaciens* and *Xanthomonas perforans*. Treatment doses (0, 25, 50, and 100) are volumes of suspended resin (μL) in 300 μL. For each resin type, bars appearing with the same letter are not different according to Tukey’s test (P < 0.05). Data shown are arithmetic means + standard errors.


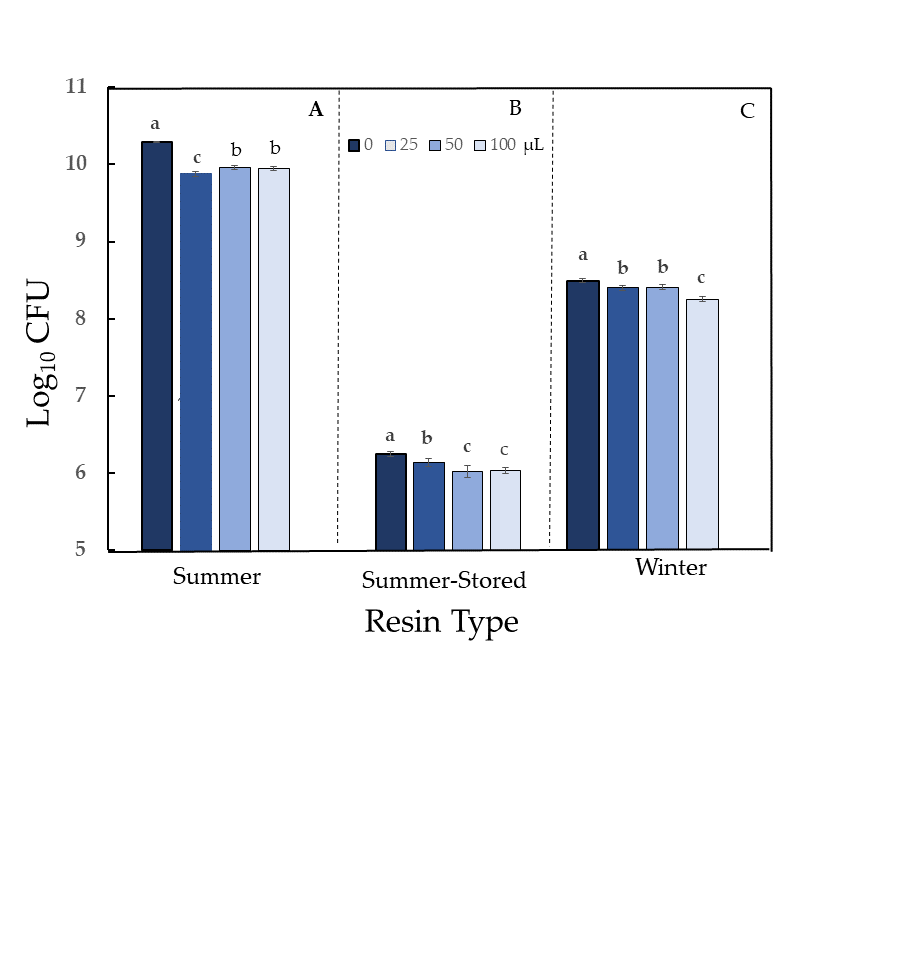
.

**Figure S4.** Impact of *Sv* resin on growth of *Escherichia coli*. (**A**) Resin collected in summer and evaluated within 72 h. (**B**) Resin collected in summer and stored at –20 °C for 6 months before evaluation. (**C**) Resin collected in winter and evaluated within 72 h. Treatment doses (0, 25, 50, and 100) are volumes of suspended resin (μL) in 300 μL. For each resin type, bars appearing with the same letter are not different according to Tukey’s test (P < 0.05). Data shown are arithmetic means + standard errors.
